# How to deprescribe esketamine in resistant depression? A point of view after first clinical uses

**DOI:** 10.1017/S204579602100072X

**Published:** 2022-01-11

**Authors:** T. Taillefer de Laportalière, A. Yrondi, A. Jullien, P. Cestac, F. Montastruc

**Affiliations:** 1 Department of Medical and Clinical Pharmacology, Centre of PharmacoVigilance and Pharmacoepidemiology, Toulouse University Hospital (CHU), Faculty of Medicine, Toulouse, France; 2 Department of Medical Psychiatry, Toulouse University Hospital (CHU), Faculty of Medicine, Toulouse, France; 3 Treatment Resistant Depression Expert Center, FondaMental, Toulouse, France; 4 ToNIC Toulouse NeuroImaging Center, University Paul Sabatier Toulouse, INSERM, Toulouse, France; 5 Department of Pharmacy, CHU de Toulouse, Toulouse, France; 6 Centre d'Investigation Clinique 1436, Team PEPSS « Pharmacologie En Population cohorteS et biobanqueS », Toulouse University Hospital, Toulouse, France

## Aims

Esketamine is the S-enantiomer of racemic ketamine. It is a non-selective, non-competitive, antagonist of the N-methyl-D-aspartate (NMDA) receptor, an ionotropic glutamate receptor. In Europe, esketamine is approved for adults with treatment-resistant major depressive disorder. Although the demonstration of the effectiveness of this medication remains uncertain (Cristea and Naudet, 2019; Turner, 2019; Gastaldon *et al*., 2020), several authors suggest an interest in certain clinical situations of resistant depression (Jauhar and Morrison, 2019; Zimmermann *et al*., 2020). Among the questions around the use of esketamine, some authors have reported concerns about the duration of esketamine prescription (Swainson *et al*., 2019). They question the long-term benefit-harm balance and in particular the risk of abuse and dependence, which could limit deprescription. At this time, there are no data to guide the frequency and duration of treatment and how to initiate discontinuation. Here, we report the first experiences with the use of esketamine in a centre specialised in resistant depression in France with the support of a multidisciplinary consultation meeting.

## Methods

In order to better monitor patients, a multidisciplinary consultation meeting was set up in November 2020. It brings together a psychiatrist specialised in resistant depression, a clinical pharmacist and a clinical psychopharmacologist. It aims to evaluate the expected benefits and risks before introducing esketamine. In our University Hospital, for each patient, a specific follow-up was set up to monitor the effectiveness of esketamine treatment via the Montgomery–Åsberg Depression Rating Scale (MADRS). We also monitor adverse drug reactions: blood pressure was measured before, then 40 and 90 min after each administration. During the post-administration follow-up, the patient was systematically asked if there were any dissociative elements or suicidal thoughts. A MADRS score is also established after each administration. Any other adverse drug reaction could be reported directly by the patient. Before each administration of esketamine, we recalled to patients the possibility of reporting any adverse drug reactions. This close follow-up starts at the initiation of the treatment and is continued until its end.

## Results

To date, a total of four patients (named patient 1, 2, 3, 4) started esketamine (Table 1). The four patients are women with no history of substance abuse. Before the introduction of esketamine, all patients had already been treated with 4–5 antidepressants (including at least one tricyclic antidepressant) and electroconvulsive therapy sessions without significant lasting clinical improvement and for some patients, with cognitive impairment. About the follow-up of the efficacy of esketamine in our four patients: the mean MADRS score of the patients before initiation of esketamine was 27.5 and all patients take a dose of 84 mg every week for 4 weeks after the induction phase then 84 mg every 2 weeks. For patient 4, MADRS decreased by 15 points after 14 weeks of esketamine treatment, for patient 3 by 11 points after 8 weeks of treatment, for patient 2 by 20 points after 36 weeks of treatment and for patient 1 by 19 points after 87 weeks of treatment. Regarding adverse drug reactions, some have been reported within 1 or 2 h after administration: blood pressure increased, dizziness and dissociative effects were spontaneously resolved after 1 h. Two serious adverse effects were reported: an increase in blood pressure (patient 1) requiring the introduction of a long-term antihypertensive drug (hydrochlorothiazide) and a suicidal episode (patient 2). The patient attempted to jump from the third floor, which required hospitalisation in psychiatry. The patient had no somatic consequences of this suicide attempt. This episode occurred 1 h after the administration of esketamine. About the discontinuation of esketamine, only patient 2 has recently attempted an abrupt discontinuation, without a dose reduction, on his own initiative after 9 months of taking it. One month later, she relapsed with an important sadness, feeling of guilt, suicidal thoughts, associated with sleep and appetite disorders, the patient has restarted inhalations of esketamine at a dose of 84 mg every 2 weeks. Patient 1 is still on esketamine after 22 months of exposure, the three attempts to space the doses at 84 mg every 3 weeks have been failed with significant fluctuations in MADRS, appearance of suicidal ideation and psychomotor retardation (after 2–3 weeks following the last administration). Patient 3 changed follow-up centre after 4 weeks but we know that she is still on esketamine, which means 7 months of exposure. Patient 4 is still being followed up after 14 weeks and again requires twice-weekly administrations because the response to esketamine is not high enough.

**Table 1.** Patient characteristics

		Patient 1	Patient 2	Patient 3	Patient 4
Patient characteristics	Age (years)	51	34	42	56
	Gender	F	F	F	F
	BMI (kg/m^2^)	35,4	20,7	26,1	34,2
	Comorbidity	HBP	–	–	–
Before esketamine initiation	Initial MADRS	34	26	20	30
	Antidepressants	9	6	4	4
During esketamine exposition	Anxiolytics	3	2	2	1
	Antipsychotics	1	1	2	1
	Mood stabilisers	0	0	0	1
	Associated antidepressants	Amytriptiline Duloxétine	Paroxétine	Sertraline Miansérine	Duloxétine

F, female; BMI, body mass index; HBP, high blood pressure; Initial MADRS, Montgomery–Åsberg Depression Rating Scale score before esketamine introduction; Antidepressants, number of antidepressants failures; Anxiolytics: number of anxiolytics associated with esketamine; Antipsychotics, number of antipsychotics associated with esketamine; Mood stabilisers, number of mood stabilisers associated with esketamine.

## Conclusion

This clinical experience on four patients allows us to discuss the question of deprescribing esketamine, especially in comparison with the data from clinical trials. First, there is no clear evaluation of the method of discontinuation of esketamine. In the European Summary of Product Characteristics (SPC), there is no information on the dosing schedule for deprescribing. In our experience, it should be noted that one patient managed to stop very quickly without a dosage reduction phase and relapsed 1 month later. Secondly, there is no robust data on prescription duration. The laboratory product information states ‘dosing frequency should be individualized to the least frequent dosing to maintain remission/response’ without specifying a duration when the SPC states ‘after depressive symptoms improve, treatment is recommended for at least 6 months’. These data are based on a consensus of ten experts (Nash *et al*., 2021). Of those, eight experts agreed on a minimum duration of 6 months. They did not give a recommendation on the maximum duration of treatment, stating that there were insufficient data on this subject. It should be noted that only 50% of the experts had experience outside the clinical trial setting. In our centre, three patients have already taken esketamine for more than 6 months after symptom improvement, whereas the majority of clinical trials assessed efficacy over only 1 month. Third, these prolonged prescription durations of more than 6 months raise questions about long-term adverse effects. In particular, we have concerns about cardiovascular side effects such as myocardial infarction or stroke. This type of effect could be expected due to the stimulation of the orthosympathic system by esketamine and the frequently found increase in blood pressure. In our centre, one of the patients required the introduction of a long-term antihypertensive drug. A recent pharmacovigilance study (Gastaldon *et al*., 2021) reviewed the adverse drug reactions reported with esketamine in the FDA Adverse Event Reporting System (FAERS) database. They found a significant association between some adverse drug reactions and esketamine such as dissociative disorders. They also found an association between esketamine and suicidal ideation, although it is difficult to make a direct association in view of the drug indication. As a reminder, patient 2 attempted suicide 1 h after administration. The authors also attempted to identify patient populations at higher risk of developing serious adverse effects with esketamine. Thus, patients receiving high doses of esketamine, associated with antidepressants, antipsychotics, benzodiazepines and mood stabilisers, would be more vulnerable. We note that our four patients fit into this category and that two of them have already had serious adverse drug reactions. Fourth, there are other effects to be monitored in the long term. Looking at the profile of ketamine, the risk of dependence and misuse (EMCDDA, 2021) and the risk of urinary adverse effects (Castellani *et al*., 2020) should be taken into account. Assessments of the risk of withdrawal syndromes, well known with ketamine, are sometimes criticised by some authors (Horowitz and Moncrieff, 2020). With ketamine, the effects of the withdrawal syndrome can include mood reduction, fatigue, changes in appetite and anxiety. All these symptoms are also present in depressions relapse.

In conclusion, our first clinical experience (even limited to four patients) does not fit into the framework of clinical trials particularly about the duration of use. To date, we lack data on the long-term safety effects and no evaluation was made to determine the duration of prescription and deprescription method. There is an urgent need to assess these issues related to long-term esketamine exposure. Only robust long-term clinical trials and real-life observational studies will allow us to better evaluate the prescription and deprescription of esketamine.

## Data Availability

The data can be available upon request from the authors.
